# Association between Nicotinamide N-Methyltransferase Gene Polymorphisms and Obesity in Chinese Han Male College Students

**DOI:** 10.1155/2017/2984826

**Published:** 2017-09-18

**Authors:** Qiong Zhou, Xiao-Juan Zhu, Jiang-Hua Li

**Affiliations:** ^1^Key Laboratory of Functional Small Organic Molecule, Ministry of Education, Jiangxi Normal University, Nanchang, China; ^2^Institute of Physical Education, Jiangxi Normal University, Nanchang, China

## Abstract

Some reports have shown that nicotinamide N-methyltransferase (NNMT) is associated with the body mass index (BMI) and energy metabolism. Here we explored the association between* NNMT* gene polymorphisms and obesity. The subjects were recruited from male Chinese Han college student. 289 of them (19 ≤ body fat percentage (BF%)) were selected as the high body fat group (HBFG), 494 of them (3 ≤ BF% < 13.5) were selected as the low body fat group (LBFG), and then a case-control study (fat versus thin) was carried out to explore the association between the* NNMT* gene polymorphism and the body composition using tagSNPs method. A tagSNP (rs10891644) in* NNMT* gene was found significantly associated with the body composition (*P* < 0.0026). At this locus, the BF% for the genotype GT, TT, and GG were 14.56 ± 8.35, 13.47 ± 8.11, and 12.42 ± 7.50, respectively, and the differences between the GT and the GG + TT were highly significant (*P* < 0.01); the OR_adjusted_ value of the GT versus (GG + TT) was 1.716 (*P*_adjusted_ = 0.002, 95% CI = 1.240–2.235). Therefore, the variation of the tagSNP, rs10891644, is significantly associated with obesity and the GT carriers are the susceptible population.

## 1. Introduction

NNMT is an enzyme which catalyzes the methylation of nicotinamide (NAM) and produces methylnicotinamide (MNA) [1, 2]. Metabolomic works have showed that levels of MNA in urine were correlated with the body mass index (BMI) significantly [3, 4]; thus indicating that NNMT is involved in the regulation of the body fat. Recently, these findings have been supported by multiple evidences. For example, serum MNA was reportedly increased in Chinese obesity population [5] and urinary MNA was found elevated in humans with obesity and type 2 diabetes (T2D), in db/db mice and in obese Zucker rats [4]. These results implicate an increased NNMT activity in obesity. The more direct evidences came from Kraus et al. [6] and Lee et al. [7]. They found that* Nnmt* knockdown caused a 47% reduction in relative fat mass of mice [6], and NNMT expression is increased in adipocytes of obese population [7].

More than two hundreds of single nucleotide polymorphisms (SNPs) have been identified within human* NNMT* gene in recent years, and two noncoding region SNPs (rs694539 and rs1941404) have been reported to be significantly associated with some related noncommunicable chronic diseases (NCD), such as hyperhomocysteinemia [8], congenital heart diseases [9], abdominal aortic diseases [10], migraine [11], nonalcoholic steatohepatitis [12], bipolar disorder [13], epilepsy [14], schizophrenia [15], and hyperlipidemia [16]. However, the association between* NNMT* gene SNPs and the body composition has not yet been reported to date.

Are there any SNPs in* NNMT* DNA sequence significantly associated with obesity? We check all the candidate genetic association studies (CGASs) on* NNMT* gene and all the genome-wide association studies (GWASs) on obesity. The results show that no CGAS concerning* NNMT* gene and obesity has been carried out to date and that no SNP in* NNMT* DNA sequence has been identified to be significantly associated with obesity in existing GWASs. Theoretically, it is not surprising that* NNMT* gene is significantly associated with obesity. As the precursor of the NAD^+^, NAM methylation can directly affect NAD^+^ levels, because NAM cannot be used in the synthesis of NAD^+^ if it is methylated by NNMT [17]. NAD^+^ is essential for the fuel oxidation in our bodies. The competition between NNMT and NAD^+^ salvage suggests that* NNMT* gene polymorphisms might affect the fuel oxidation and the storage of fat. Then, why no SNP in* NNMT* gene has been identified to be significantly associated with obesity in GWASs?

In fact, GWASs usually apply strictest genome-wide significance criterion to overcome type I error which makes the false negative discoveries inevitable, therefore a CGAS is still necessary to check whether there are any* NNMT* SNPs significantly associated with the body composition. Moreover, the most existing GWASs used SNP data from HapMap Project, but many more SNPs have been sequenced in 1000 Genome Project nowadays, so it is possible that some significant SNPs in* NNMT* gene were not detected in GWASs.

To test this speculation, a tagSNP based CGAS was carried out in this investigation. Within a candidate gene, the number of SNPs may be large, but direct analysis of all SNPs is inefficient, because the genotypes at many of these loci are strongly correlated. It is a common method to select the maximally informative set of SNPs (tagSNPs) to analyze in a CGAS, thus all known SNPs either are directly assayed or highly associated with a tagSNP [18]. Here, we identified 19 tagSNPs (including rs694539 and rs1941404) across the whole* NNMT* gene DNA sequence using Haploview software (Haploview 4.2) in the first place and then performed a case-control (fat versus thin) study to explore the association between these tagSNPs and obesity.

## 2. Subjects and Methods

### 2.1. Subjects and Grouping

783 subjects were selected from Chinese Han male college students (aged from 17 to 23 years). According to their body fat percentage (BF%), the selected subjects were divided into two groups: the high body fat group (HBFG, 19 ≤ BF%, *n* = 289) and the low body fat group (LBFG, 3 ≤ BF% < 13.5, *n* = 494). The inclusion criteria were free of any diagnosed diseases (especially the anorexia, bulimia nervosa, and the diseases in the digestive system), without partiality for a particular kind of food and without exercise habit. The local ethics committee of Jiangxi Normal University approved this investigation, and the written informed consent was given to all the participants. This study conforms to the latest revision of the Declaration of Helsinki.

### 2.2. Body Component Measurement

Body component was measured using bioimpedance measurement with an X-SCAN PLUS body composition analyzer (X-SCAN PLUSII, Jawon Medical Co., Ltd., South Korea). Measurements were performed in the morning with empty stomachs.

### 2.3. SNP Selection and Genotyping

As mentioned above, there are many SNPs in* NNMT* gene. To determine the investigation of loci, a tagSNP approach was used [18]. With the criteria (MAF > 0.10 and *r*^2^ > 0.8), nineteen tagSNPs were selected using Haploview software (Haploview 4.2) from the known SNP data in the Chinese Han population (CHB + CHS), which were downloaded from the database, 1000 Genomes Project (http://browser.1000genomes.org). Genomic DNA was extracted from blood samples with DNA extraction kits (Promega, USA). The gene sequence was downloaded from the database of National Center for Biotechnology Information (NCBI). Both probes and primers were designed with online software Primer 3 (http://bioinfo.ut.ee/primer3-0.4.0). Genotypes of the SNPs were detected by polymerase chain reaction-ligase detection reaction (PCR-LDR) [17]. Ten percent of the PCR-LDR reactions were performed in duplicate to check the reliability of the genotyping, and more than 99.5% of them had the matching results. Additionally, Sanger sequencing method was used to genotype the significantly associated tagSNP (rs10891644) of 30 samples randomly selected, and 100% had the matching results with the PCR-LDR method.

### 2.4. Statistics

The frequency distributions of genotype and allele and Hardy–Weinberg equilibrium (HWE) were analyzed online (http://analysis.bio-x.cn). The HWE tests only performed in the control group. Four genetic models provided by Zintzaras and Santos [19] and two classification logistic regressions were used in the genotype effects analyses. The mean values were compared with one-way ANOVA with IBM SPSS Statistics 20.0 (SPSS Inc., Chicago, IL, USA). *P* value < 0.05 was considered statistically significant. Bonferroni correction was performed for the analysis of the genotype and allele frequency distributions, and the corrected significance level was *P* < 0.0026. The *P* values and the odds ratios were adjusted for the age in the analysis of the genotype effects.

## 3. Results and Discussions

### 3.1. The Distributions of Alleles and Genotypes of the 19 Tag SNPs

The allele and genotype distributions of the 19 tagSNPs are shown in the Table 1. Among these SNPs, rs10891644 was the only one significantly associated SNP after Bonferroni correction (*P* < 0.0026) and qualified with HWE test (*P* > 0.05). At this locus, the HBLG exhibited a higher allele T frequency and a higher genotype GT frequency than the LBFG did. Thus the rs10891644 variation was focused in the rest analyses and the other SNPs were not analyzed any longer.

### 3.2. Genotype Effects and Genetic Models of rs10891644 Variation

Genetic models (dominant, recessive, additive, and codominant) are often used in genotype effect analyses. However, these models are not totally independent. To avoid hash of these models, a whole solution and the degree of dominance (*h*) were offered by Zintzaras and Santos [19]. As shown in the Table 2, chances of subjects of the genotypes GG, GT, and TT to be the HBFG were 31%, 44%, and 35%, respectively. Two classification logistic regression analyses suggested that the dominant model and the codominance model were both significant (*P*_adjusted_ < 0.05) after the adjustment for age, and the adjusted degree of dominance (*h*_adjusted_) was less than −1 (|*h*| > 1 indicates that the phenotype of heterozygotes lies outside the phenotypical range of both homozygotes). Based on the *P*_adjusted_ values, these results indicate that the possible inheritance modes of the rs10891644 variation are dominant or codominant. However, the *P*_adjusted_ value of the additive model (GG versus TT) demonstrated that the difference between the homozygous GG and TT was not significant (*P*_adjusted_ > 0.05), which denied the dominant mode, because in the dominant mode the difference between the homozygous genotypes must be significantly different. To determine whether the inheritance mode is overdominant, the *h*_adjusted_ value has been calculated, and the *h*_adjusted_ = 2.57 strongly suggests that the inheritance mode of the rs10891644 variation is overdominant. Overdominant inheritance is a condition in genetics where the phenotype of the heterozygote lies outside the phenotypical range of both homozygotes, which usually is described as heterosis, wherein heterozygous individuals have advantages in the natural selection. In this study, the OR_adjusted_ value of the GT versus (GG + TT) was 1.716, which means that the chance of the GT being of HBFG is 1.716 times that of the homozygous (GG + TT), thus indicating that the heterozygous individuals (GT carriers) are the susceptible population to obesity. It is worth noting that although many diseases are associated with obesity nowadays, fat storage might be very helpful to enhance survival chances when lack of food in the long history of mankind. Therefore, it may be a result from the natural selection that the heterozygous individuals (GT carriers) are the susceptible population to obesity.

**Figure 1:**
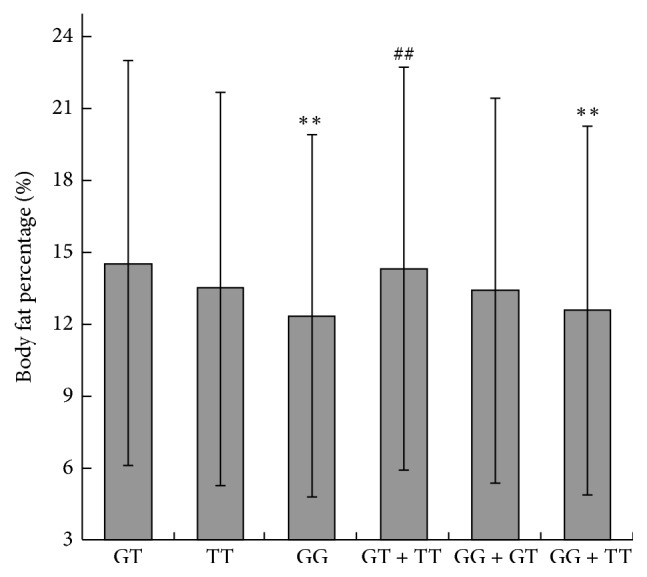
Comparisons of the body fat percentage between the different genotypes. GT, the genotype GT (*n* = 336); TT, the genotype TT (*n* = 82); GG, the genotype GG (*n* = 354); GT + TT, the genotypes (GT + TT) (*n* = 418); GG + GT, the genotypes (GG + GT) (*n* = 690); GG + TT, the genotypes (GG + TT) (*n* = 436). ^*∗∗*^*P* < 0.01 compared with GT; ^##^*P* < 0.01 compared with GG. Error bars, ± standard deviation.

To further justify the genotype effects of the rs10891644 variation shown in Table 2, we compared the BF% between the subjects with different genotypes. As shown in Figure 1, the BF% of the GT, TT, and GG carriers were 14.56 ± 8.35, 13.47 ± 8.11, and 12.42 ± 7.50, the highest was the GT carriers followed by the TT and the GG carriers, and there were highly significant differences (*P* < 0.01) between the GT and the GG carriers, between the GT and the GG + TT carriers (codominant model), and between the GT + TT and the GG carriers (dominant model), respectively, while there were no significant differences between the GG and TT carriers (additive model) and between the TT and the GG + GT carriers (recessive model) (*P* > 0.05). These results further demonstrated that the body composition is significantly affected by the rs10891644 variation, and the inheritance mode of the rs10891644 variation is overdominant.

As mentioned above, although no* NNMT* gene SNP had been reported to be significantly associated with obesity before this paper, numerous reports have confirmed the roles of NNMT in regulation of the body composition. Among individuals, NNMT activity varies fivefold and has a bimodal frequency distribution in livers [20], but when the cDNAs of individuals with high and low NNMT activity were compared, no sequence differences were seen [21]. Thus the differences of phenotypes are due to the differences at transcriptional level and not because of* NNMT* SNPs in the coding regions [21].* NNMT* gene is highly polymorphic in humans, and most of which are in the noncoding regions of this gene. The tagSNP (rs10891644), which was found significantly associated with obesity in this study, is also in the noncoding region (5′ near gene). Therefore, it presumably affects the transcription of* NNMT* gene, thus causing the genetic risk for fat storage.

The existing reports have shown that NMT plays roles in the regulation of energy metabolism [6, 22, 23] and the body composition [3–7]. Therefore it is reasonable that* NNMT* gene polymorphism is associated with obesity. However, besides the genetic factor, many other factors are also related to obesity, such as race, gender, age, diet, exercise, and the gut microbiome [23–29]. To maximally eliminate the influences from other factors, we recruited the subjects from Chinese Han male college students, who were free of any diagnosed diseases (especially the anorexia, bulimia nervosa, and the diseases in the digestive system), without partiality for a particular kind of food and without exercise habit, and did the adjustment for age. The limitations of this investigation are that all participants were not entirely on the same controlled diet and that the influences of gut microbiome could not be eliminated.

In summary, for the first time we found that a tagSNP (rs10891644) in* NNMT* gene is significantly associated with obesity and the heterozygous individuals (GT carriers at this locus) are the susceptible population. Although the precise mechanism of the regulation process still needs further investigation, our findings suggest that the variant of a tagSNP (rs10891644) in* NNMT* gene is involved in the etiopathology of obesity in Chinese Han male college students.

## Figures and Tables

**Table 1:** The distribution of alleles and genotypes of the 19 tag SNPs.

SNPs	Allele frequency			*P*	Genotype frequency			HWE	*P*
rs2511153	Case	C: 352 (0.63)	T: 206 (0.37)	0.708	CC: 108 (0.39)	CT: 57 (0.44)	TT: 20 (0.16)		0.827
	Ctrl.	C: 625 (0.64)	T: 351 (0.36)		CC: 199 (0.41)	CT: 227 (0.47)	TT: 62 (0.13)	0.83	
rs505978	Case	A: 307 (0.54)	C: 261 (0.46)	0.668	AA: 80 (0.28)	AC: 254 (0.52)	CC: 57 (0.20)		0.909
	Ctrl.	A: 544 (0.55)	C: 442 (0.45)		AA: 145 (0.29)	AC: 66 (0.50)	CC: 94 (0.19)	0.36	
rs694539	Case	A: 192 (0.34)	G: 374 (0.66)	0.613	AA: 30 (0.11)	AG: 132 (0.47)	GG: 121 (0.43)		0.861
	Ctrl.	A: 347 (0.35)	G: 639 (0.65)		AA: 55 (0.11)	AG: 237 (0.48)	GG: 201 (0.41)	0.23	
rs12285641	Case	C: 349 (0.62)	T: 217 (0.38)	0.817	CC: 102 (0.36)	CT: 145 (0.51)	TT: 36 (0.13)		0.820
	Ctrl.	C: 585 (0.61)	T: 373 (0.39)		CC: 174 (0.36)	CT: 237 (0.50)	TT: 68 (0.14)	0.38	
rs11214926	Case	A: 154 (0.27)	G: 410 (0.73)	0.871	AA: 18 (0.06)	AG: 118 (0.42)	GG: 146 (0.52)		0.810
	Ctrl.	A: 273 (0.28)	G: 713 (0.72)		AA: 28 (0.06)	AG: 217 (0.44)	GG: 248 (0.50)	0.03	
rs7109984	Case	C: 502 (0.89)	T: 64 (0.11)	0.221	CC: 224 (0.79)	CT: 54 (0.19)	TT: 5 (0.02)		0.407
	Ctrl.	C: 829 (0.87)	T: 129 (0.14)		CC: 359 (0.75)	CT: 111 (0.23)	TT: 9 (0.02)	0.90	
rs10891644	Case	G: 370 (0.64)	T: 206 (0.36)	0.029^*∗*^	GG: 111 (0.39)	GT: 148 (0.51)	TT: 29 (0.10)		0.002^*∗∗*^
	Ctrl.	G: 674 (0.70)	T: 294 (0.30)		GG: 243 (0.50)	GT: 188 (0.39)	TT: 53 (0.11)	0.07	
rs55675450	Case	A: 88 (0.16)	G: 470 (0.84)	0.368	AA: 5 (0.02)	AG: 78 (0.28)	GG: 196 (0.70)		0.056
	Ctrl.	A: 138 (0.14)	G: 842 (0.86)		AA: 17 (0.04)	AG: 104 (0.21)	GG: 369 (0.75)	0.01	
rs2244175	Case	A: 274 (0.48)	G: 292 (0.52)	0.147	AA: 64 (0.23)	AG: 146 (0.52)	GG: 73 (0.26)		0.337
	Ctrl.	A: 515 (0.52)	G: 471 (0.48)		AA: 132 (0.27)	AG: 251 (0.51)	GG: 110 (0.22)	0.65	
rs2847492	Case	A: 198 (0.35)	G: 368 (0.65)	0.162	AA: 33 (0.12)	AG: 132 (0.47)	GG: 118 (0.42)		0.366
	Ctrl.	A: 380 (0.39)	G: 606 (0.62)		AA: 72 (0.15)	AG: 236 (0.48)	GG: 185 (0.38)	0.82	
rs2852432	Case	C: 336 (0.59)	T: 232 (0.41)	0.209	CC: 94 (0.33)	CT: 148 (0.52)	TT: 42 (0.15)		0.286
	Ctrl.	C: 551 (0.56)	T: 435 (0.44)		CC: 153 (0.31)	CT: 245 (0.50)	TT: 95 (0.19)	0.86	
rs4646335	Case	A: 326 (0.57)	T: 242 (0.43)	0.387	AA: 89 (0.31)	AT: 148 (0.52)	TT: 47 (0.17)		0.666
	Ctrl.	A: 588 (0.60)	T: 398 (0.40)		AA: 169 (0.34)	AT: 250 (0.51)	TT: 74 (0.15)	0.24	
rs3819100	Case	A: 282 (0.50)	G: 284 (0.50)	0.597	AA: 70 (0.25)	AG: 142 (0.50)	GG: 71 (0.25)		0.825
	Ctrl.	A: 505 (0.51)	G: 481 (0.49)		AA: 126 (0.26)	AG: 253 (0.51)	GG: 114 (0.23)	0.55	
rs2256292	Case	C: 234 (0.41)	G: 338 (0.59)	0.417	CC: 50 (0.18)	CG: 134 (0.47)	GG: 102 (0.36)		0.526
	Ctrl.	C: 382 (0.39)	G: 602 (0.61)		CC: 71 (0.14)	CG: 240 (0.49)	GG: 181 (0.37)	0.55	
rs2301128	Case	A: 81 (0.15)	G: 477 (0.86)	0.071	AA: 3 (0.01)	AG: 75 (0.27)	GG: 201 (0.72)		0.051
	Ctrl.	A: 111 (0.11)	G: 867 (0.89)		AA: 8 (0.02)	AG: 95 (0.19)	GG: 386 (0.79)	0.44	
rs1941404	Case	C: 265 (0.47)	T: 301 (0.53)	0.472	CC: 59 (0.21)	CT: 147 (0.52)	TT: 77 (0.27)		0.762
	Ctrl.	C: 443 (0.45)	T: 543 (0.55)		CC: 94 (0.19)	CT: 255 (0.52)	TT: 144 (0.29)	0.32	
rs2155806	Case	C: 59 (0.11)	T: 499 (0.89)	0.200	CC: 1 (0.00)	CT: 57 (0.20)	TT: 221 (0.79)		0.387
	Ctrl.	C: 125 (0.13)	T: 853 (0.87)		CC: 4 (0.01)	CT: 117 (0.24)	TT: 368 (0.75)	0.11	
rs1941399	Case	A: 102 (0.18)	C: 464 (0.82)	0.425	AA: 4 (0.01)	AC: 94 (0.33)	CC: 185 (0.65)		0.152
	Ctrl.	A: 194 (0.20)	C: 792 (0.80)		AA: 19 (0.04)	AC: 156 (0.32)	CC: 318 (0.65)	0.98	
rs4646337	Case	A: 479 (0.86)	G: 79 (0.14)	0.935	AA: 201 (0.72)	AG: 77 (0.28)	GG: 1 (0.00)		0.102
	Ctrl.	A: 841 (0.86)	G: 137 (0.14)		AA: 362 (0.74)	AG: 117 (0.24)	GG: 10 (0.02)	0.88	

HWE, Hardy–Weinberg equilibrium; Case, the high body fat group; Ctrl., the low body fat group. HWE, *P* value of Hardy–Weinberg equilibrium test on the control group; the values of allele and genotype are the number of individuals (frequency); ^*∗*^*P* < 0.05; ^*∗∗*^*P* < 0.01.

**Table 2:** Genotype effects and genetic models of the rs10891644 variation.

Model	Genotype	HBFG	LBFG	OR (95% CI)	Adjusted OR (95% CI)	*P* (*P*_adjusted_)	*h* (*h*_adjusted_)
Recessive	TT	29 (0.35)	53 (0.65)	0.911 (0.564, 1.469)	0.932 (0.538, 1.614)	0.701 (0.802)	2.83 (2.57)
	(GT + GG)	259 (0.38)	431 (0.62)				
Dominant	(TT + GT)	177 (0.42)	241 (0.58)	1.608 (1.195, 2.163)	1.669 (1.188, 2.345)	0.002 (0.003)^*∗∗*^	
	GG	111 (0.31)	243 (0.69)				
Additive	TT	29 (0.35)	53 (0.65)	1.198 (0.723, 1.985)	1.239 (0.695, 2.208)	0.484 (0.468)	
	GG	111 (0.31)	243 (0.69)				
Codominant	GT	148 (0.44)	188 (0.56)	1.664 (1.240, 2.235)	1.716 (1.220, 2.413)	0.001 (0.002)^*∗∗*^	
	(TT + GG)	140 (0.32)	296 (0.68)				

HBFG, the high body fat group; LBFG, the low body fat group; the values of HBFG and LBFG are the number of individuals (ratio) from different genotypes; OR, odds ratio; CI, confidence interval; *h* (dominance degree) = ln⁡(OR_co_)/ln⁡(OR_a_), OR_co_, OR of the codominant model, OR_a_, OR of the additive model, |*h*| > 1 indicates that the phenotype of the heterozygotes lies outside the phenotypical range of both homozygotes; Adjusted, adjustment for age. ^*∗∗*^*P* < 0.01.
